# Supplementary material for: The Copper(II)-Thiodiacetate (tda) Chelate as Efficient Receptor of N9-(2-Hydroxyethyl)Adenine (9heade): Synthesis, Molecular and Crystal Structures, Physical Properties and DFT Calculations of [Cu(tda)(9heade)(H2O)]·2H2O
Source: Molecules. 2023 Aug 2;28(15):5830. doi: 10.3390/molecules28155830 (PMC10420684; doi:10.3390/molecules28155830)

Supplementary Material for

Article

The Copper(II)-Thiodiacetate (tda) Chelate as Efficient Receptor of N9-(2-Hydroxyethyl)Adenine (9heade): Synthesis, Molecular and Crystal Structures, Physical Properties and DFT Calculations of $[Cu(tda)(9heade)(H_2O)]\cdot 2H_2O$

Carmen Rosales-Martínez [1], Antonio Matilla-Hernádez [1], Duane Choquesillo-Lazarte [2], Antonio Frontera [3], Alfonso Castiñeiras [4] and Juan Niclós-Gutiérrez [1,*]

[1] Department of Inorganic Chemistry, Faculty of Pharmacy, University of Granada, 18071 Granada, Spain; carmenromar@correo.ugr.es (C.R.-M.); amatilla@ugr.es (A.M.-H.)
[2] Laboratorio de Estudios Cristalográficos, IACT, CSIC-Universidad de Granada, Avda. de las Palmeras 4,
18100 Armilla-Granada, Spain; duane.choquesillo@csic.es
[3] Departament de Química, Universitat de les Illes Balears, Crta. de Valldemossa km 7.5,
07122 Palma de Mallorca, Spain; toni.frontera@uib.es
[4] Department of Inorganic Chemistry, Faculty of Pharmacy, University of Santiago de Compostela,
15782 Santiago de Compostela, Spain; alfonso.castineiras@usc.es
* Correspondence: jniclos@ugr.es

Table S1. Selected bond lengths [Å] and angles [°] for $[Cu(tda)(9heade)(H_2O)]\cdot 2H_2O$.

Table S2. Hydrogen bonds for $[Cu(tda)(9heade)(H_2O)]\cdot 2H_2O$ [Å and °]

Figures S1. FT-IR spectra

Figure S2. Electronic spetrum (diffuse reflectance)

Figure S3. TGA analysis

Figure S4. Comparison powder X-ray diffraction patterns

Figure S5. ESR spectra.

Table S1. Selected bond lengths [Å] and angles [°] for [Cu(tda)(9heade)(H_2O)]·$2H_2O$.

Cu(1)-O(4)	1.933(2)
Cu(1)-O(1)	1.962(2)
Cu(1)-N(21)	2.025(2)
Cu(1)-O(8)	2.262(2)
Cu(1)-S(1)	2.3625(8)
Cu(1)-O(2)[a]	3.060(4)
O(4)-Cu(1)-O(1)	175.04(10)
O(4)-Cu(1)-N(21)	96.16(9)
O(1)-Cu(1)-N(21)	87.56(9)
O(4)-Cu(1)-O(8)	86.36(10)
O(1)-Cu(1)-O(8)	96.03(9)
N(21)-Cu(1)-O(8)	102.95(9)
O(4)-Cu(1)-S(1)	87.22(7)
O(1)-Cu(1)-S(1)	88.77(7)
N(21)-Cu(1)-S(1)	173.58(7)
O(8)-Cu(1)-S(1)	82.67(5)
O(4)-Cu(1)-O(2)[a]	70.77(10)
O(1)-Cu(1)-O(2)[a]	105.90(9)
N(21)-Cu(1)-O(2)[a]	92.04(9)
O(8)-Cu(1)-O(2)[a]	153.95(8)
S(1)-Cu(1)-O(2)[a]	83.90(6)

Symmetry transformations used to generate equivalent atoms:
a = x-1,y,z.

Table S2. Hydrogen bonds for [Cu(tda)(9heade)(H_2O)]·$2H_2O$ [Å and °].

D-H···A	d(D-H)	d(H···A)	d(D···A)	∠(DHA)
O(1)-H(1B)...O(8)[a]	0.85	1.86	2.699(3)	168.2
O(1)-H(1A)...O(9)[b]	0.85	1.79	2.662 (3)	163.7
O(2)-H(2C)...O(4)	0.85	2.50	3.108 (4)	129.7
O(2)-H(2C)...O(5)	0.85	1.86	2.691 (4)	164.7
O(2)-H(2D)...O(5)[c]	0.85	1.98	2.816 (3)	167.8
O(3)-H(3A)...O(9)[b]	0.85	1.98	2.819 (3)	169.9
O(3)-H(3B)...N(23)[a]	0.85	2.11	2.918 (4)	157.7
O(32)-H(32)...O(3)	0.82	1.93	2.744 (3)	170.8
N(26)-H(26A)...O(2)[a]	0.86	1.96	2.757 (4)	154.0
N(26)-H(26B)...N(27)[d]	0.86	2.18	2.980 (4)	154.1
C(2)-H(2A)...O(32)[e]	0.97	2.35	3.297 (4)	165.2
C(6)-H(6B)...O(32)[e]	0.97	2.53	3.443 (4)	156.4
C(22)-H(22)...O(1)[f]	0.93	2.52	3.412 (4)	161.2
C(22)-H(22)...O(8)	0.93	2.61	3.282 (4)	129.6

Symmetry transformations used to generate equivalent atoms:

a = x-1,y,z; b = -x+1,-y+1,-z+1; c = -x+2,-y+1,-z+2; d = -x,-y,-z+2; e = x,y+1,z; f = x+1,y,z

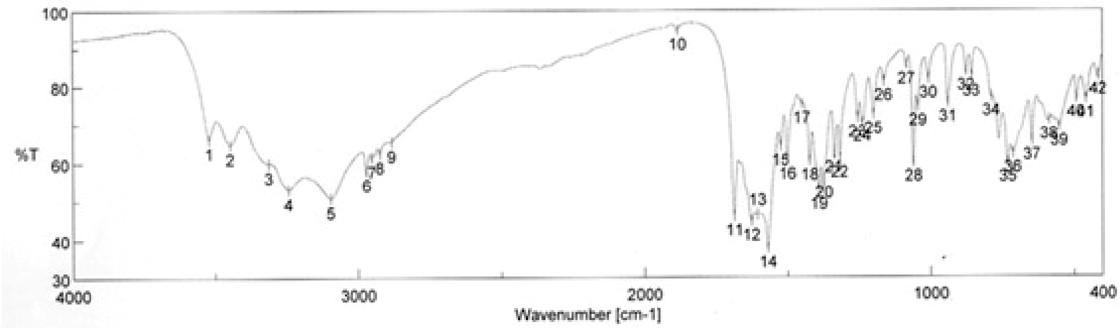

[Result of Peak Picking]

No.	Position	Intensity	No.	Position	Intensity	No.	Position	Intensity
1	3521.38	66.0202	2	3446.17	64.6513	3	3313.11	59.7551
4	3242.72	52.928	5	3097.12	50.7111	6	2969.84	57.806
7	2951.52	61.3051	8	2924.52	62.3599	9	2884.02	65.2647
10	1883.15	94.5863	11	1686.44	45.9874	12	1627.63	44.7063
13	1606.41	46.3469	14	1570.74	37.5623	15	1525.42	64.0976
16	1500.35	60.5881	17	1450.21	75.1749	18	1423.21	60.349
19	1391.39	52.7427	20	1373.07	55.6248	21	1339.32	62.3386
22	1319.07	61.0095	23	1255.43	71.5281	24	1239.04	70.3587
25	1200.47	72.415	26	1164.79	81.0214	27	1084.76	85.3574
28	1059.69	59.9967	29	1044.26	74.4588	30	1007.62	81.7111
31	940.128	75.4933	32	876.488	83.6893	33	858.168	82.1281
34	787.779	77.0193	35	730.889	59.7875	36	713.533	62.1769
37	647.001	65.4159	38	586.254	70.798	39	551.542	69.0303
40	492.723	76.5139	41	458.975	76.4116	42	415.585	82.4419

(a)

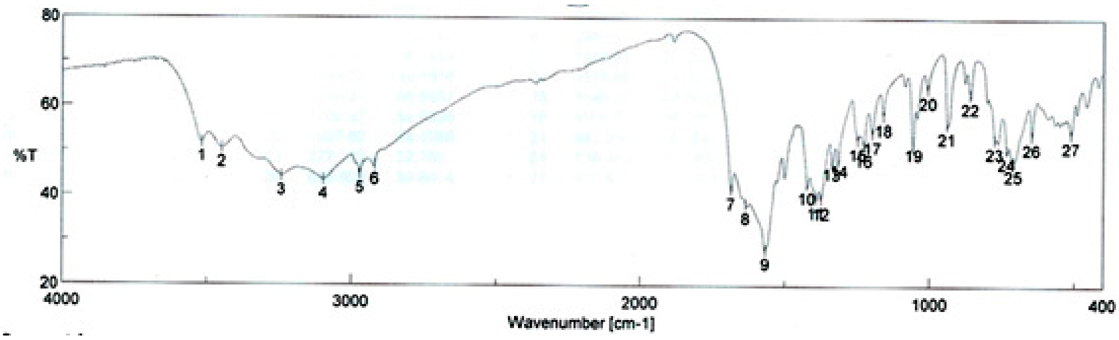

(b)

Figure S1a. Minimally ground sample of compound 1.b.Ground sample of compound 1.

Figure S2. Electronic spetrun (diffuse reflectance)

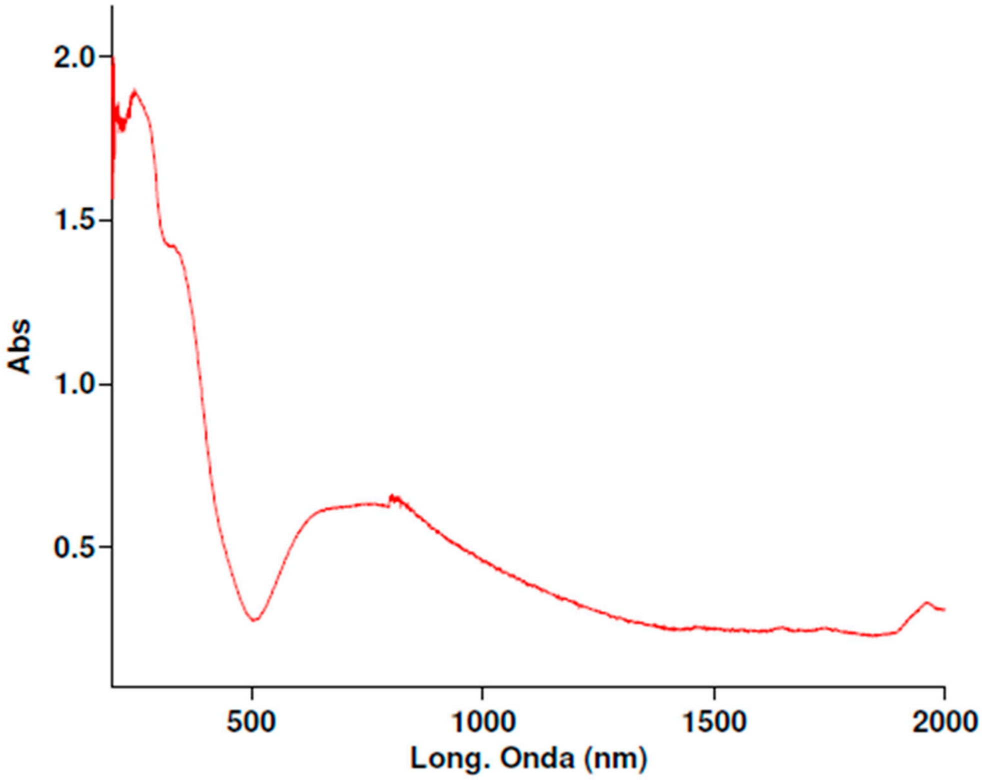

Figure S3. TGA analysis. Three FT-IR spectra of the evolved gases during the TGA of compound **1.**

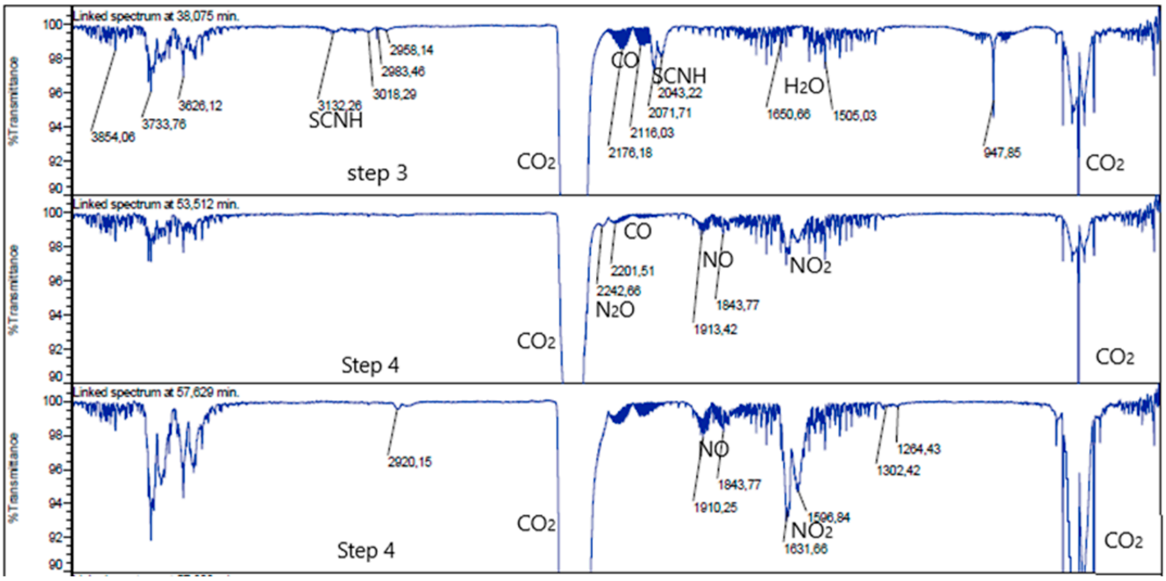

Figure S4. Comparison between experimental (blue) (collected using Phillips and room temperature) and calculated powder X-ray diffraction patterns for the complex 1. The two diffractograms are similar, confirming that the bulk crystalline sample consists of a single phase.

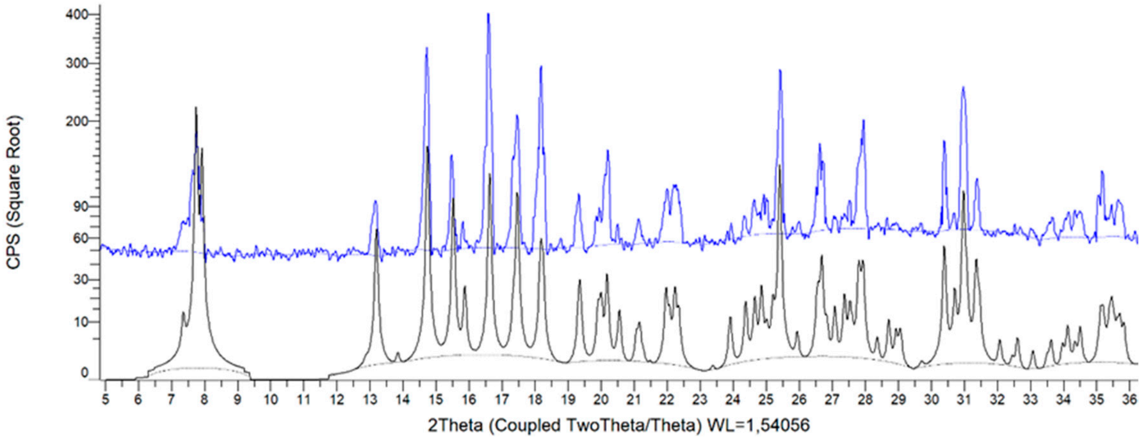

Figure S5. ESR spectra.

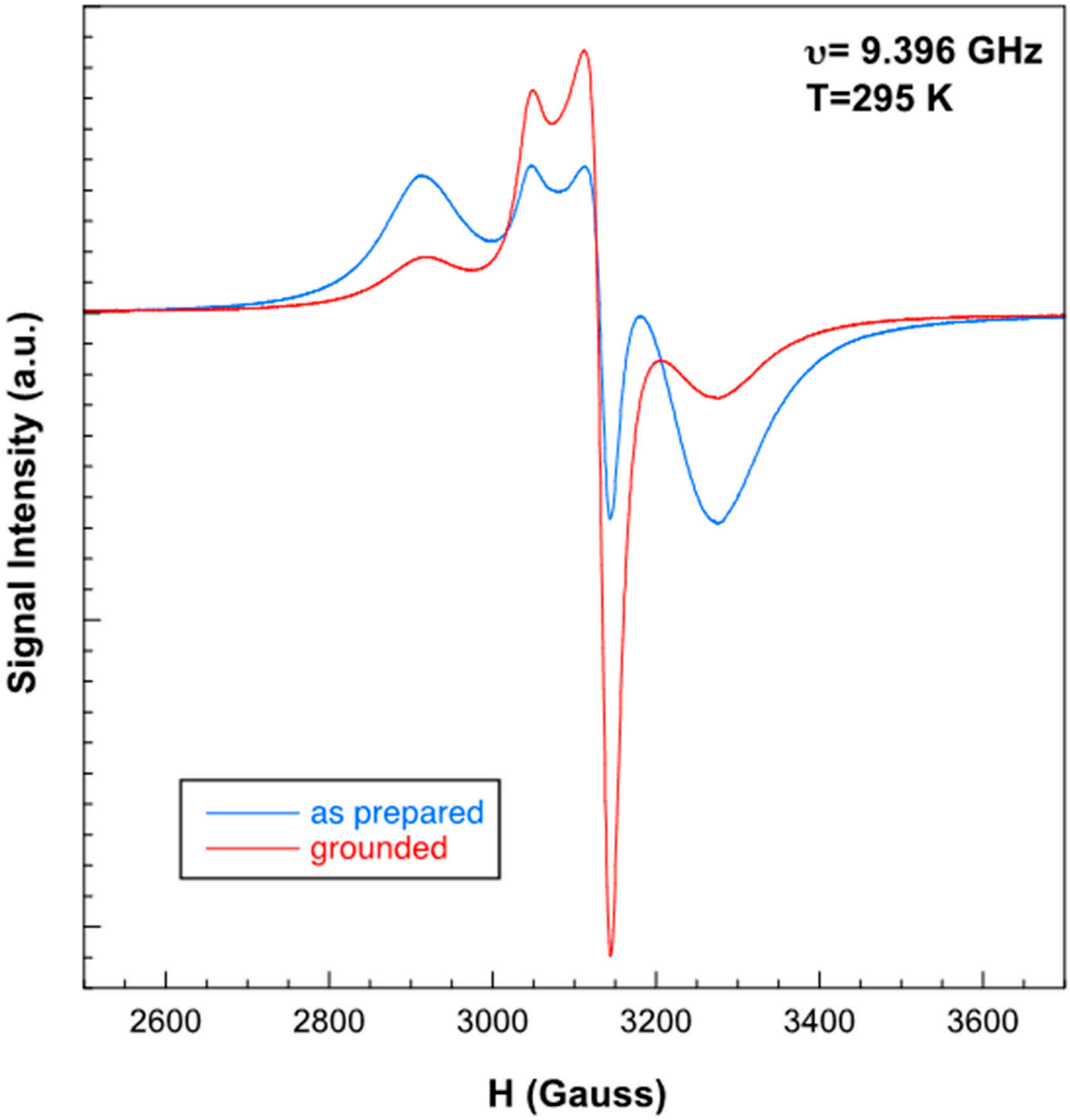

Supplement: Supplementary file 1 [file molecules-28-05830-s001.zip › molecules-2474285-supplementary.pdf]
